# Attitudes among healthcare professionals to the reporting of adverse drug reactions in Nepal

**DOI:** 10.1186/2050-6511-14-16

**Published:** 2013-03-08

**Authors:** Santosh KC, Pramote Tragulpiankit, Sarun Gorsanan, I Ralph Edwards

**Affiliations:** 1 Faculty of Pharmacy, Mahidol University, Bangkok, Thailand; 2 Bir Hospital, Kathmandu, Nepal; 3 Faculty of Pharmacy, Siam University, Bangkok, Thailand; 4 Uppsala Monitoring Centre, Uppsala, Sweden

**Keywords:** Adverse drug reaction, Attitudes, Healthcare professional, Nepal

## Abstract

**Background:**

Healthcare professional’s knowledge and attitudes to adverse drug reaction (ADR) and ADR reporting play vital role to report any cases of ADR. Positive attitudes may favour ADR reporting by healthcare professionals. This study was aimed to investigate the attitudes towards and ways to improve adverse drug reaction (ADR) reporting among healthcare professionals working at four Regional Pharmacovigilance Centres (RPCs) of Nepal.

**Methods:**

A cross sectional study was done by survey using a self-administered structured questionnaire. The questionnaire was distributed to 450 healthcare professionals working at four RPCs.

**Results:**

The overall response rate was 74.0%. There were 74.8% of healthcare professionals who had seen patient experiencing an ADR; however, only 20.1% had reported. Reporting form not available (48.1%) and other colleagues not reporting ADR cases (46.9%) would significantly discourage the ADR reporting among healthcare professionals working at four RPCs. Healthcare professionals perceived that seriousness of the reaction (75.6%); unusual reaction (64.6%); reaction to new product (71.2%); new reaction to existing product (70.2%); and confidence in diagnosis of ADR (60.8%) were important factors on the decision to report ADR. Awareness among healthcare professionals (85.9%), training (76.0%), collaboration (67.0%), and involve pharmacist for ADR reporting (63.1%) were mostly recognized ways to improve reporting. Regular newsletter on current awareness in drug safety (71.2%), information on new ADR (65.8%), and international drug safety information (64.0%) were the identified feedbacks they would like to receive from the Nepal pharmacovigilance programme.

**Conclusion:**

Healthcare professionals working at four RPCs of Nepal have positive attitudes towards ADR reporting. Awareness among healthcare professionals, training and collaboration would likely improve reporting provided they would receive appropriate feedback from the national pharamcovigilance programme.

## Background

Spontaneous reporting system (SRS) still remains as the most common method to report adverse drug reaction (ADR) even though under reporting is estimated higher than 90–95% [1-4]. Healthcare professionals are the primary reporter of the ADR cases either to national centre or to Pharma Company. There are different factors which encourage healthcare professionals to report ADRs. Among all, healthcare professionals’ knowledge about and attitudes towards ADR and ADR reporting debate more frequently as an influential factors [5-8]. Reporting of each and every cases of ADR is important; however, reporting of previously unknown ADR, rare ADR and serious unlabeled ADR is more important to generate new signal and new knowledge. Healthcare professionals are reluctant to report ADR when the ADR is common, too trivial and uncertainty about the association [9-11]. But it is interesting that some healthcare professionals especially doctors report ADR because of their professional interest to inform others [12]. Overall, knowledge about and attitudes towards ADR plays vital role in terms of ADR reporting.

In Nepal, ADR reporting is not mandatory for healthcare professionals. The Department of Drug Administration (DDA), the national drug regulatory authority, was established in 1979 to enforce the Drug Act 1978. After its establishment it had banned several drugs and its combinations on the ground of irrational combination, potential toxicity, doubtful efficacy, and potential for irrational use [13]. Though the need of pharmacovigilance has been identified early; however, it is started several years after its establishment. The DDA took the initiatives to set up a pharmacovigilance programme in 2002. In 2004, DDA was designated as a National Pharmacovigilance Centre (NPC). Two years later, it became full member of World Health Organization (WHO) collaborating Centre for International Drug Monitoring. Immediately after its establishment, NPC facilitated the operation of Regional Pharmacovigilance Centre (RPC) in different part of the country. Currently there are six RPCs operating in the country based on Kathmandu, Lalitpur, Pokhara and Biratnagar. Though the NPC is encouraging the RPCs to report more ADR, the current reporting trend suggests high under reporting. There was only total of 304 ADR cases reported during the year 2006 to 2009 by four RPCs [14]. Therefore, the purpose of this study is to investigate the knowledge and attitudes of healthcare professionals to report ADR working at four RPCs of Nepal and to suggest possible ways to improve the ADR reporting based on the findings. The findings of knowledge about ADR and ADR reporting among healthcare professionals will be presented elsewhere.

## Methods

### Study design and setting

This study was conducted in the four RPCs of Nepal. The four RPCs were Manipal Teaching Hospital (MTH), Pokhara, Tribhuvan University Teaching Hospital (TUTH), Kathmandu, Nepal Medical College Hospital (NMCH), Kathmandu and KIST Medical College Hospital (KISTMCH), Lalitpur. All those RPCs are teaching hospital in nature.

A cross sectional study was done by survey using a self-administered structured questionnaire. The attitude components of the questionnaire are presented in Additional file 1. There were 450 self-administered structured questionnaires distributed to all potential healthcare professionals (doctors, nurses and pharmacists) working at four RPCs. The questionnaire was structured to obtain the demographics of healthcare professionals, factors discouraging ADR reporting, factors that they perceived may influence reporting, ways to improve ADR reporting and feedbacks they would like to receive from NPC. Questionnaire was designed to five level likert scale (1 = strongly disagree and 5 = strongly agree) and single choice. The questionnaire was attached with the covering letter, which had aimed to provide the information of the research to the participants. The participant information sheet contained the objective of the research, the number of participants expected to include in the research, the way to response the questionnaire, their right to decide about whether or not to participate in the research and confidentiality of the response. The questionnaire so designed was tested for content validity by consensus of the expert’s panel comprising Prof. Ralph Edwards and Assist. Prof. Pramote Tragulpiankit. Objectivity test was done by distributing to 10 of the principal investigator’s colleagues and instructors of Mahidol University, Bangkok, Thailand. The comments made were incorporated and questionnaire was modified accordingly. Finally, pilot study was conducted at two hospitals of Nepal, which were Alka Hospital Pvt. Ltd., Lalitpur and Civil Service Hospital, Kathmandu, for the reliability of the questionnaire. There were 50 questionnaires randomly distributed among doctors, nurses and pharmacists working at two hospitals. The reliability of the questionnaire was evaluated by calculating Cronbach alpha. The alpha score for the attitudes towards ADR was calculated 0.81 and was considered good.

### Data collection

The self-administered structured questionnaires were distributed among healthcare professional through different departments of the four RPCs. The first response was collected within 3 weeks of the distribution. After that reminder was sent to all respondents with apologize to the ones who have already answered the questionnaire. The second response was collected within 3 weeks of the reminder. Targeted follow up was also done after this reminder to the respondents by personal visit or telephone call. This study was approved by Mahidol University, Faculty of Dentistry/ Faculty of Pharmacy, Institutional Review Board (MU-DT/PY-IRB) and, Institutional Review Board of the four hospitals before starting the data collection.

### Statistical analysis

The SPSS statistical programme for Windows, version 17.0 was used for the analysis of the data. The coded data was systematically verified and checked for errors. Results were presented as mean ± standard deviation for quantitative variables and number with percentage or graphic presentation for categorical variables, where applicable. Percentage on each category, median and mode was presented for the likert scale. The chi-square test was performed to find out the association between ADR occurrence and ADR reporting among healthcare professionals. The comparison of the attitudes among different category of healthcare professionals was analyzed by Kruskal Wallis test. Significance level of P < 0.05 was used, where the test was relevant.

## Results

Out of 450 questionnaires distributed, 333 were received back with an overall response rate of 74.0%. There were 128 males and 201 females. Among the respondents, 4 did not mention about their gender and profession. There were 162 doctors, 135 nurses and 32 pharmacists. Among the respondents, 66.4% were in the age group 21–30 years and 67.9% of them had experiences of 1–5 years followed by 13.2% who had experiences of 6–10 years. The mean age and the experience were 29.5 years and 5.4 years, respectively. The characteristic features of the respondents are shown in Table 1.

**Table 1:** Demography details and characteristic features of the respondents

**Category**	**Sub-category**	**Number (%)**
**Gender**	Male	128 (38.4)
	Female	201 (60.4)
	Data missing	4 (1.2)
**Age (years)**	Up to 20	9 (2.7)
	21–30	221 (66.4)
	31–40	69 (20.7)
	41–50	19 (5.7)
	51–60	3 (0.9)
	Above 60	6 (1.8)
	Mean	29.5
	Minimum	19
	Maximum	72
	Data missing	6 (1.8)
**Professional qualification**	Doctor	162 (48.6)
	Nurse	135 (40.5)
	Pharmacist	32 (9.6)
	Data missing	4 (1.2)
**Work experience (years)**	Less than 1	9 (2.7)
	1–5	226 (67.9)
	6–10	44 (13.2)
	11–15	10 (3.0)
	16–20	11 (3.3)
	21–25	7 (2.1)
	26–30	4 (1.2)
	Above 30	6 (1.8)
	Mean	5.4
	Minimum	0
	Maximum	40
	Data missing	16 (4.8)
**Category**	Sub-category*	**Number (%)**
**Doctor**	MD/MS	70 (21.0)
	MBBS	86 (25.8)
	MDS	3 (0.9)
	BDS	2 (0.6)
**Nurse**	MN	2 (0.6)
	BN	36 (10.8)
	PCL	97 (29.1)
**Pharmacist**	PhD and Master	9 (2.7)
	BPharm	6 (1.8)
	PCL/Diploma	17 (5.1)
**Country of undergraduate study**	Nepal	163 (75.8)
	India	26 (12.1)
	Bangladesh	11 (5.1)
	China	5 (2.3)
	Philippines	2 (1.6)
	Russia	1 (0.5)
	Ukraine	1 (0.5)
	Data missing	6 (2.8)

*Note: *MD*: Doctor of medicine, *MS*: Master of surgery, *MBBS*: Bachelor of medicine and surgery, *MDS*: Master of dental surgery, *BDS*: Bachelor of dental surgery, *MN*: Master in nursing, *BN*: Bachelor in nursing, *PCL*: Proficiency certificate level, *PhD*: Doctor of Philosophy, *BPharm*: Bachelor of Pharmacy.

Two hundred and forty six healthcare professionals (74.8%) had seen patient experiencing an ADR. Among them, 82.7% of doctors, 67.4% of nurses and 65.6% of pharmacists had seen ADR during their routine work (P = 0.005). In contrast, only 66 respondents (20.1%) had ever reported an ADR to the pharmacovigilance centre/unit of their hospital. There were 21.6% of doctors, 17.0% of nurses and 25.0% of pharmacists who had ever reported ADR (P = 0.440). The details are shown in Table 2. There were 38.3% of doctors, 40.7% of nurses, and 28.1% of pharmacists provided reasons for ADR not reported. Among the respondents, 28.4% of doctors, 26.7% of nurses, and 9.4% of pharmacists were unaware about the existence of PV centre/unit in the hospital.

**Table 2:** Experience of ADR occurrence and ADR reporting among healthcare professionals

**Category**	**Profession**	**Yes (%)**	**No (%)**	P-value*
Even seen any patient experiencing an ADR	**Total**	**246 (74.8)**	**83 (25.2)**	
	Doctor	134 (82.7)	28 (17.3)	P=0.005
	Nurse	91 (67.4)	44 (32.6)	
	Pharmacist	21 (65.6)	11 (34.4)	
Ever reported an ADR to the Pharmacovigilance Centre/ Unit of his/her hospital	**Total**	**66 (20.1)**	**263 (79.9)**	
	Doctor	35 (21.6)	127 (78.4)	P=0.440
	Nurse	23 (17.0)	112 (83.0)	
	Pharmacist	8 (25.0)	24 (75.0)	

* Chi-square test.

Two hundred and forty five respondents (75.6%) agreed (score 4 or 5 on the likert scale) on seriousness of the reaction, 230 (71.2%) agreed on reaction to new product, 226 (70.2%) agreed on new reaction to existing product, 208 (64.6%) agreed on unusual reaction, and 196 (60.8%) agreed on confidence in diagnosis of ADR as an important factors on the decision to report ADR. In contrast, ADR reporting form not available 155 (48.1%) and other colleagues not reporting ADR cases 151 (46.9%) were the major discouraging factors. Among the respondents, 167 (51.8%) disagreed (score 1 or 2 on the likert scale) on ADR reporting as a guilt of causing patient harm and 165 (51.2%) disagreed on ambition to publish case report personally as a factor discouraging ADR reporting. Similarly, 151 (46.9%) disagreed on ADR reporting will generate extra work, 141 (43.8%) disagreed on fear of legal liability, 141 (43.8%) disagreed on belief of only safe drugs are marketed, and 129 (40.1%) disagreed on lack of time to actively look for an ADR (Table 3).

**Table 3:** Factors discouraging ADR reporting

**Factors**	**n**	**Response (%)**					**Median**	**Mode**
		**1**^**a**^	**2**^**b**^	**3**^**c**^	**4**^**d**^	**5**^**e**^		
Concern that the report may be wrong	323	68 (21.1)	58 (18.0)	85 (26.3)	64 (19.8)	48 (14.9)	3.0	3
Lack of time to fill in a report and a single unreported case may not affect ADR database	322	65 (20.2)	54 (16.8)	86 (26.7)	74 (23.0)	43 (13.4)	3.0	3
Not confident to decide whether or not an ADR has occurred	322	72 (22.4)	48 (14.9)	91 (28.3)	70 (21.7)	41 (12.7)	3.0	3
Lack of time to actively look for an ADR while at work	322	74 (23.0)	55 (17.1)	77 (23.9)	70 (21.7)	46 (14.3)	3.0	3
Fear of legal liability by reporting adverse reaction	322	87 (27.0)	54 (16.8)	88 (27.3)	51 (15.8)	42 (13.0)	3.0	3
Concern that a report will generate an extra work	322	97 (30.1)	54 (16.8)	87 (27.0)	56 (17.4)	28 (8.7)	3.0	1
Belief that only safe drugs are marketed	322	74 (23.0)	67 (20.8)	74 (23.0)	66 (20.5)	41 (12.7)	3.0	1*
Think that you may have caused a patient harm	322	99 (30.7)	68 (21.1)	76 (23.6)	46 (14.3)	33 (10.2)	2.0	1
Ambition to publish case report personally	322	99 (30.7)	66 (20.5)	89 (27.6)	39 (12.1)	29 (9.0)	2.0	1
Reporting forms are not available when needed	322	40 (12.4)	46 (14.3)	81 (25.2)	71 (22.0)	84 (26.1)	3.0	5
Other colleagues are not reporting ADR cases	322	54 (16.8)	43 (13.4)	74 (23.0)	79 (24.5)	72 (22.4)	3.0	4

^a^ 1 = strongly disagree.

^b^ 2 = moderately disagree.

^c^ 3 = neutral.

^d^ 4 = moderately agree.

^e^ 5 = strongly agree.

* More than one mode exists, the lowest is presented.

Two hundred and eighty six respondents (85.9%) suggested awareness among healthcare professionals, training for healthcare professionals 253 (76.0%), collaboration among other healthcare professionals 223 (67.0%), involve pharmacists for ADR reporting 210 (63.1%), and make reporting a professional obligation 184 (55.5%) as a possible ways to improve ADR reporting in the context of Nepal. Majority of the respondents would like to receive feedbacks from the national pharmacovigilance programme. The identified mode of feedbacks were regular newsletter on current awareness on drug safety 237 (71.2%), information of new ADR by newsletter 219 (65.8%), international drug safety information 213 (64.0%), annual national statistics 196 (58.9%), and individual response to report 146 (43.8%).

## Discussion

This study identified the attitudes towards ADR and ADR reporting among healthcare professionals working at four RPCs of Nepal. Among six RPCs, we did not include two RPCs in this study as they recognized after this study was started. This study found that healthcare professionals have positive attitudes towards ADR and ADR reporting. The overall response rate of 74.0% was acceptable. The higher percentage of female respondents compared to male is because of nurses as a segmented group included in this research. Nursing practitioners in Nepal are female only. While looking at experiences and ages of the respondents, mostly the young and beginner healthcare professionals were participated in this research. In Nepal, the clinical role of pharmacist is in infancy, mostly involved in dispensing and counseling, rather than direct pharmaceutical care. Two hundred and six healthcare professionals participated in this research had only first degree qualification to practice. One hundred and sixty three had perused the qualification in Nepal. In the curricula of Bachelor of Medicine and Surgery (MBBS) and Bachelor of Pharmacy (BPharm) in Nepal, the content of ADR and ADR reporting is not adequately covered. However, as a part of MBBS course in some institutions students are trained to ADR reporting and causality assessments [15]. But in UK, majority of the medical schools have included yellow card scheme in the undergraduate syllabuses and most of them assess student knowledge on the scheme [16]. The healthcare professionals, doctors, nurses and pharmacists, are the main reporter of the ADR case which they encountered on their routine work in general; however, involvement of nurses as a reporter is not well accepted by hospital physicians [17]. In Nepal, nurses are allowed to report ADR; however, they are not encouraged enough to report.

Different studies reported that all the ADRs encountered by healthcare professionals during their work are never reported [8,11,17-19], even though, majority of them felt ADR reporting is important in principle. This study also showed the same trend in terms of ADR encountered and ADR reporting. Two hundred and forty six (74.8%) healthcare professionals who had seen patient experiencing an ADR. However, only 66 (20.1%) of them had reported to the pharmacovigilance centre/unit of their hospital. But in the countries where ADR monitoring system is well established for example UK, France, Netherland and Sweden the ADR reporting rates among physicians estimate 40–70% [6-8,17,20]. This might be because ADR reporting is mandatory in all those countries. In 2006, the reporting rate in Sweden was 563 per million inhabitants [17]. The main reason for not reported ADR cases in this study were healthcare professionals did not know about existence of pharmacovigilance centre/unit in their hospitals. The other reasons for not reporting ADR were, reactions were not serious, did not know how to report, reported to concern doctors, did not think necessary to report.

This study showed positive attitudes to ADR reporting among healthcare professionals working at four RPCs of Nepal. Out of 11 discouraging factors to report ADR provided to rate, most had neutral response. The median of 9 factors was 3. Due to lack of practice of ADR reporting most of the respondents might have chosen neutral response. We found that reporting form not available and other colleagues not reporting ADR cases would significantly influence the ADR reporting among healthcare professional. Reporting form not available identified as a discouraging factor to report ADRs by other studies too [19,21,22]. On the other hand, lack of time to actively look for an ADR while at work, fear of legal liability, ADR reporting will generate extra work, belief of only safe drugs are marketed, think that they may have caused patient harm and ambition to publish case report personally were not significant factors to discourage ADR reporting among healthcare professionals. Even though, 119 (36.0%) of the healthcare professionals agreed on lack of time to actively look for an ADR while at work and 107 (33.2%) believed that only safe drug are marketed. This is suggestive for train healthcare professionals by including ADR reporting and causality assessment in undergraduate syllabuses. This study showed that healthcare professionals working at four RPCs perceived that seriousness of the reaction, unusual reaction, reaction to new product, new reaction to existing product and, confidence in diagnosis of ADR are important factors on the decision to report ADR. Seriousness, unusual reaction, reaction to new product and certainty were also identified as important factors to report ADR among physicians in different studies [7,8,17,18].

Awareness among healthcare professionals, collaboration among other healthcare professionals and training for healthcare professionals were the highly suggested ways to improve ADR reporting. Healthcare professionals believed that making ADR reporting, a professional obligation and involved pharmacists for ADR reporting can also improve ADR reporting. Previous studies have also identified ADR reporting as a professional obligation [21,23]. In some countries for example Sweden, France, ADR reporting by healthcare professionals is compulsory, even though, the impact to counter under-reporting is still controversial [24]. ADR reporting as a professional obligation will have moral binding to healthcare professionals and ethical issues. Studies have shown that pharmacist involvement can improve the number and quality of ADR reports along with substantial role in maintenance of drug safety monitoring programme [25-27]. The feedbacks they would like to receive from the national pharmacovigilance programme as regular newsletters on current awareness in drug safety, information on new drug adverse reactions by newsletters, annual national statistics and international drug safety information can be incorporate in a single newsletter. DDA is publishing Drug Bulletin of Nepal (BDN) on a regular basis [28]. The scope of the bulletin is wide. It can be updated with a regular column related to national and international drug safety information, even though; it has regular information related to current awareness in drug safety. The feedback from the national centre ensures two way communications between healthcare professionals and the national centre. In Sweden, feedback letters along with result of causality assessment of the reported ADR case is sent to the reporter concerned [17]. This is supposed to be one of the possible reasons of the high reporting rate in Sweden and elsewhere.

There are some limitations of this study. First, the sample does not represent the whole population of healthcare professionals of Nepal as it was conducted only on the four RPCs of Nepal, where the ADR monitoring is already in place. Second, questionnaires were distributed through different departments of the hospitals, responding by the aid of relevant books/publications or contemporary colleagues could not be excluded.

## Conclusion

This study showed that healthcare professionals working at four RPCs of Nepal have positive attitudes about ADR reporting; however, the reporting culture is not well developed as reflected by the huge gap between the ADR encountered and ADR reporting trend among healthcare professionals. The unavailability of ADR reporting form and colleague’s negative reporting nature are significantly discouraging them to report ADRs. However, awareness among healthcare professionals, training and collaboration would likely improve ADR reporting provided they would receive appropriate feedbacks from the national pharmacovigilance programme preferably as regular newsletter on current awareness in drug safety, information on new ADR and international drug safety information.

## Abbreviations

ADR: Adverse drug reaction; RPC: Regional Pharmacovigilance Centre; SRS: Spontaneous reporting system; DDA: Department of Drug Administration; NPC: National Pharmacovigilance Centre; WHO: World Health Organization; MTH: Manipal Teaching Hospital; TUTH: Tribhuvan University Teaching Hospital; NMCH: Nepal Medical College Hospital; KISTMCH: KIST Medical College Hospital; MU-DT/PY-IRB: Mahidol University, Faculty of Dentistry/ Faculty of Pharmacy, Institutional Review Board; MBBS: Bachelor of medicine and surgery; BPharm: Bachelor of Pharmacy; PCL: Proficiency certificate level; MD: Doctor of medicine; MS: Master of surgery; MDS: Master of dental surgery; BDS: Bachelor of dental surgery; MN: Master in nursing; BN: Bachelor in nursing; PhD: Doctor of Philosophy, DBN, Drug Bulletin of Nepal; DTC: Drug and Therapeutic Committe

## Competing interests

The authors have no competing interests to report.

## Authors’ contributions

SKC and PT designed the study and draft questionnaire. SKC collected data of the study. PT, SG and IRE suggested the data analysis and sequence of draft manuscript. All the authors participated in the critical review of the draft manuscript, editing and approval for submission.

## Pre-publication history

The pre-publication history for this paper can be accessed here:

http://www.biomedcentral.com/2050-6511/14/16/prepub

## Supplementary Material

Additional file 1 Questionnaire for evaluating healthcare professionals’ attitudes towards ADR reporting in Nepal. Click here for file
