# Supplementary material for: Attitudes among healthcare professionals to the reporting of adverse drug reactions in Nepal
Source: BMC Pharmacol Toxicol. 2013 Mar 8;14:16. doi: 10.1186/2050-6511-14-16 (PMC3599543; doi:10.1186/2050-6511-14-16)
Supplement: Additional file 1 — Questionnaire for evaluating healthcare professionals’ attitudes towards ADR reporting in Nepal. [file 2050-6511-14-16-S1.doc]

**Attitudes among healthcare professionals to the reporting of adverse drug reactions in Nepal**

**Questionnaire for attitude components**

**___________________________________________________________**

**Details of the responder**

Age: …………. Gender: Male/Female Date: ………………

Professional qualification: ………………. Department: ………………………

Country of undergraduate study: ………..Years of practice: ………Specialty: ………

Role: o Administrator o Practitioner o Other: ……………

**___________________________________________________________**

1. Have you ever seen any patient experiencing an adverse drug reaction?

a. Yes

b. No

If ***“Yes”***, how often____________________________________________________

If ***“ No”,*** please give reason _____________________________________________

2. Have you ever reported an adverse drug reaction to the Pharmacovigilance Centre /Unit of your hospital?

a. Yes

b. No

If ***“Yes”***, how often____________________________________________________

If ***“ No”,*** please give reason _____________________________________________

3. Which factors do you think ***discourage*** to report adverse drug reactions?

Please select the number according to your opinion. 5= strongly agree, 4= moderately agree, 3= neutral, 2= moderately disagree, 1= strongly disagree

| S. No. | Factors | 1 | 2 | 3 | 4 | 5 |
| --- | --- | --- | --- | --- | --- | --- |
| a. | Concern that the report may be wrong |  |  |  |  |  |
| b. | Lack of time to fill in a report and a single unreported case may not affect ADR database |  |  |  |  |  |
| c. | Not confident to decide whether or not an ADR has occurred |  |  |  |  |  |
| d. | Lack of time to actively look for an ADR while at work |  |  |  |  |  |
| e. | Fear of legal liability by reporting adverse reaction |  |  |  |  |  |
| f. | Concern that a report will generate an extra work |  |  |  |  |  |
| g. | Belief that only safe drugs are marketed |  |  |  |  |  |
| h. | Think that you may have caused a patient harm |  |  |  |  |  |
| i. | Ambition to publish case report personally |  |  |  |  |  |
| j. | Reporting forms are not available when needed |  |  |  |  |  |
| k. | Other colleagues are not reporting |  |  |  |  |  |

4. Which factors do you think is important in your decision to report an adverse drug reaction?

Please select the number according to your opinion. 5= strongly agree, 4= moderately agree, 3= neutral, 2= moderately disagree, 1= strongly disagree

| S. No. | Factors | 1 | 2 | 3 | 4 | 5 |
| --- | --- | --- | --- | --- | --- | --- |
| a. | Seriousness of the reaction |  |  |  |  |  |
| b. | Unusual reaction |  |  |  |  |  |
| c. | Reaction to a new product |  |  |  |  |  |
| d. | New reaction to a existing product |  |  |  |  |  |
| e. | Confidence in the diagnosis of ADR |  |  |  |  |  |

5. What are the possible ways to improve adverse drug reaction reporting in the contest of Nepal? (You can choose more than one option)

| S. No. | Ways to improve reporting | Tick |
| --- | --- | --- |
| a. | Awareness among healthcare professional |  |
| b. | Collaboration among other healthcare professional |  |
| c. | Make reporting a professional obligation |  |
| d. | Training to the healthcare professional |  |
| e. | Involve pharmacist for ADRs reporting |  |
|  | Please suggest other possible ways of improving ADR reporting (please list as many points as possible) |  |

6. What feedback would you like from the Nepal Pharmacovigilance Programme?

(You can choose more than one option)

| S. No. | Feedback | Tick |
| --- | --- | --- |
| a. | Individual response to report |  |
| b. | Information on new drug adverse reactions by Newsletter |  |
| c. | Regular Newsletter on current awareness in drug safety |  |
| d. | Annual national statistics |  |
| e. | International drug safety information |  |
| f. | Please suggest other feedback |  |

*** Thank you for your co-operation.***
